# Inflammatory Related Reactions in Humans and in Canine Breast Cancers, A Spontaneous Animal Model of Disease

**DOI:** 10.3389/fphar.2022.752098

**Published:** 2022-02-11

**Authors:** Domenico Tricarico, Anna Sara Convertino, Irsida Mehmeti, Girolamo Ranieri, Francesco Leonetti, Carmelo Laface, Nicola Zizzo

**Affiliations:** ^1^ Department of Pharmacy‐Pharmaceutical Sciences, University Aldo Moro, Bari, Italy; ^2^ Department of Pharmaceutical Science, Faculty of Pharmacy, “Catholic University Our Lady of Good Counsel”, Tirana, Albania; ^3^ Interventional and Medical Oncology Unit, Department of Pathology National Cancer Research Centre, IRCCS Istituto Tumori Giovanni Paolo II, Bari, Italy; ^4^ Section of Veterinary Pathology and Comparative Oncology, Department of Veterinary Medicine, University of Bari “Aldo Moro”, Bari, Italy

**Keywords:** breast cancer, immunoistochemistry, canine animal model, inflammation, pharmacovigilance

## Abstract

Inflammatory cells are emerging markers in various cancers in human trials. The relationship between the inflammatory cells response, cancer grade, and progression has been investigated experimentally in a spontaneous canine model of breast cancer and in the unselected population (18–64 years.o.) under anti-HER2 treatments that represent the most prevalent population in this cancer type. The canine data (N samples = 101) were collected retrospectively for diagnosis in our regional area and evaluated by immunohistochemistry and haemato-chemistry. The inflammatory and immune-related adverse reactions (ADR) in humans were evaluated using EudraVigilance. The “Proportional Reporting Ratio” (PRR) of the mabs was calculated for each ADR with values >2 indicative of high risk. In dogs, we found elevated immunostaining of CD68-macrophages in the lymph node of the aggressive cancer G3 and infiltrating CD20+-lymphocyte. A high density of CD20 + lymphocytes was observed in G1 and a decrease in the density was observed with the histological degree of the tumors. The animals with the sample in G1 showed reduced serum platelet and neutrophil count and elevated lymphocytes and the opposite in severely affected animals. Inflammatory reactions with edema, skin reactions, extravasation, loss of effectiveness, and platelet count decrease (PRR > 13) were found with trastuzumab emtansine in humans, in the absence of immune system reactions. Trastuzumab i.v.-s.c. showed immune system reactions, loss of effectiveness, intolerances with drug withdrawal, technological issues (PRR > 7), and neutrophil count decrease reports. These reactions were less frequently reported for pertuzumab i.v. Case reports of platelet and neutrophil count decrease were not associated with disease progression with a better outcome in humans as in canine breast cancer. Therefore, infiltrating CD68-macrophages are associated with G3, while infiltrating CD20^+^ and elevated serum lymphocytes in parallel with reduced platelet and neutrophil count play a favorable role in human and canine breast cancer.

## Introduction

Cancer is the second leading cause of death after cardiovascular disease. Considering the entire population, and taking care of non-melanoma skin cancers, the most frequent tumors are those of the udder and rectum, followed by that of the lung and prostate and in the case of skin cancers, the five most frequently diagnosed cancers among men are prostate cancer, lung cancer, colorectal cancer, bladder cancer, kidney cancer, and urinary tract cancer; among women, breast cancer, colorectal cancer, lung cancer, thyroid cancer, and cervical cancer. In cancer patients, there is a serious impairment of the immune system, as cancer cells develop mechanisms to evade immuno-surveillance (Smyth et al., 2001). Immunotherapy has been developed that uses the patient’s immune system, or certain components stimulating the body’s immune system made it more effective in recognizing and destroying cancer cells. This strategy is based on the fact that inflammatory and immune cells play a role in different cancers.

Oncology immunotherapy can be divided into active and passive immunotherapy. Active immunotherapy mainly includes cancer vaccines based on dendritic cells and T-cell transfer: these treatment strategies modify the patient’s dendritic cells or T-cells, and then re-infused and the same later; passive, instead, including monoclonal antibodies, checkpoint inhibitors, cytokines, and bispecific T-cell engagers to influence T-cell proliferation, angiogenesis, and pathways to activation. Inflammatory responses with the involvement of the cell infiltrates can be expected in human breast cancer patients under treatment with anti-HER2 monoclonal antibodies (mabs) combined with chemotherapy. Cardiovascular, inflammatory, and immune-related reactions were associated with the use of anti-HER2 mabs alone or in combination with anthracycline and chemotherapy in interventional clinical trials requiring cardiologist intervention (Martin et al., 2009; Albini et al., 2010; Yousif and Al-Amran, 2011; Bowles et al., 2012; ElZarrad et al., 2013; Zamorano et al., 2016; Lobenwein et al., 2021). Among these, trastuzumab i.v.-s.c. (HERCEPTIN Roche), trastuzumab emtansine i.v. (KADCYLA Baxter), pertuzumab i.v. (PERJETA Roche), and trastuzumab deruxtecan i.-v. (ENHERTU Daiichi Sankyo Europe) cause adverse reactions in an elevated percentage of patients that increase as a function of age, comorbidities, and cancer progression (Bowles et al., 2012; Serrano et al., 2012). Case reports showed the first case of trastuzumab emtansine-associated pleural and pericardial effusions in a patient with breast cancer in the absence of other etiologies was reported that was associated with severe inflammatory reactions due to trastuzumab emtansine (Lombardi et al., 2021). A higher rate of peripheral neuropathy and liver disfunction was also reported following trastuzumab emtansine treatments in the patients that were associated with inflammatory reactions (Battisti et al., 2020).

Five trastuzumab biosimilars have been approved by the F.D.A. and European Medicines Agency: trastuzumab-dkst (Mylan GmbH, Steinhausen, Switzerland), trastuzumab-pkrb (Celltrion Inc., Incheon, Republic of Korea), trastuzumab-dttb (Samsung Bioepis Co., Ltd., Incheon, Republic of Korea), trastuzumab-qyyp (Pfizer Ireland Pharmaceuticals, Cork, Ireland) and trastuzumab-anns (Amgen Inc., Thousand Oaks, CA, United States) (Barbier et al., 2019; Miller and Schwartzberg, 2019).

The role of inflammatory cell infiltrate in cancer is emerging; T-lymphocytes have been associated with breast cancer (Macchetti et al., 2006; Kuroda et al., 2021), seminoma (Hadrup et al., 2006), melanoma (Taylor et al., 2007), colorectal (Galon et al., 2006), cervical (Piersma et al., 2007), and ovarian (Zhang et al., 2003) in human. Biomarkers of inflammation such as the neutrophil-lymphocyte ratio (NLR) and platelet-lymphocyte ratio (PLR), are possible surrogate markers of outcome in various cancers including breast cancer with low NLR and PLR associated with better outcomes in different cancers (Hee Yeon et al., 2019; Al Jarroudi et al., 2021). Neutrophils play an important role in immune response since they affect both innate immunity and cell signaling in the adaptive immune response, and they are also a major factor in the suppression of cancer progression. Inflammation that begins with the passage of neutrophils from the circulation to tissues is an essential element not only for infections but also for immune response in cancer. Neutrophils act on the elements of the complement system and the adaptive immune system, thereby strengthening the inflammatory response (Hee Yeon et al., 2019; Kaytaz Tekyol et al., 2021). Platelets can also be involved in the inflammatory response by increasing angiogenesis or by providing growth factor release. Substances released from platelets are required for the activation of endothelial cells surrounding the vascular tract in inflammation. The presence of different subtype T lymphocytes in the tumor microenvironment (tumor-infiltrating lymphocytes) has also been associated with a positive prognosis in various malignancies including triple-negative breast cancer (Graziano et al., 2019; Hee Yeon et al., 2019; Kuroda et al., 2021). The role of neutrophils is however controversial; neutrophils have been associated with high breast cancer risk in a population-based case-control study conducted in Spain (Gago-Dominguez et al., 2018). Inflammatory cells have also an emerging role in canine breast cancer with some similarities with human disease in terms of inflammatory response and the cytotoxic role of the CD8^+^ lymphocyte (Carvalho et al., 2014); much less is known about the role of the CD20 + lymphocyte in canine breast cancer that represents a spontaneous animal model of disease. Mammary tumors are the most common neoplasms in female dogs as in humans. It is evident a greater frequency of this tumor in Europe than in the United States (Sorenmo, 2003). Age and race are risk factors in humans and canine breast cancer and the most prevalent case are hormone-related. The familial case were associated with BRCA-1 and BRCA-2 in human whose mutations predispose to the onset of breast and ovarian cancer and which are estimated to be responsible for about 80% of hereditary syndromes affecting these two organs and two-thirds of hereditary breast cancers alone in humans. Similarly, in dogs, these genes were associated with familial cases (Rivera et al., 2009). Despite several similarities with human disease, the incidence of this tumor is three times that in humans suggesting additional risk factors (Pastor et al., 2020).

Tumor-infiltrating lymphocytes, in particular CD8^+^ cells and CD20^+^ cells, were strongly associated with survival in different carcinomas (Nielsen et al., 2012; Edin et al., 2019; Hee Yeon et al., 2019; Kuroda et al., 2021) and canine glioma (Krane et al., 2021), but the role of CD20^+^ B cells was not reported in canine breast cancer. The CD20^+^ lymphocyte is proposed to play a role in the immune response against cancers in association with lymphocytes such as CD8^+^. In addition, inflammatory-based reactions data of these mabs from spontaneous reports and their correlation with the outcome are however poorly described.

In this work, we investigated the relationship between the inflammatory cells response, cancer grade, and progression in the canine breast cancer biopsies collected in our regional area for diagnostic purposes and in the unselected population (18–64 years.o.) under anti-HER2 treatments. The role of the inflammatory cells in breast cancer was validated using for the first time to our knowledge data extracted from the EudraVigilance database that contains reports of inflammatory and immune-related reactions (ADR) of patients under anti-HER2 treatments in the adult unselected population. These patients represent the most prevalent population affected by breast cancer.

## Methods

### Cancer Biopsies

A total specimen from 101 dog females with spontaneous neoplasia mammary has been recruited into the archives of Pathology and Comparative Oncology to the Department of Medicine Veterinary (Bari-Italy) for diagnosis with the written consent of the animal owners with Ethical approvement from Univ. of Bari. The gland mammary has been removed with radical mastectomy or regional with lymph nodes. The animal samples did not show the presence of metastasis.

Surgical samples fixed in 10% buffered formalin and stained with Hematoxylin-Eosin were classified histologically following Goldschmidt classification (2011) (Goldschmidt et al., 2011) and Peña for tumor grading system (2013) (Patruno et al., 2009; Zizzo et al., 2010; Pena et al., 2013; Rasotto et al., 2017).

Specimens sections were cut (4 μm thick), placed on poly-L-lysine coated glass slides, subsequently, deparaffinized in xylene and dehydrated. Subsequently immunohistochemically stained according to the labeled streptavidin avidin-biotin (LSAB) method (Ioachim, 2008).

### Tissue Sections and Immunostaining

To detect the macrophages (TAMs) and lymphocytes, the sections were immersed in citrate buffer (0.1, pH 0.6) and subjected to microwave irradiation for 15 min. All the sections were treated for 30 min with 0.3% hydrogen peroxide, then in methanol for 12 min to quench endogenous peroxidase activity (12 ml H2O2 in 400 ml of methanol). After washing three times for 5 min each with phosphate-buffered saline (PBS), the sections were blocked by soaking for 20 min at room temperature in PBS containing 1% bovine serum albumin. The blocked sections were incubated overnight at 48°C anti-mouse primary antibodies for monoclonal human antibody anti-CD20 B-LY1 (SANTA CRUZ BIOTECHNOLOGY, INC.) diluted 1/50, for 40 min and Monoclonal Mouse Anti-Human CD68 Clone KP1 (Dako, Glostrup, Denmark) diluted 1/50 then thoroughly washed in 0.05 M buffered with Tris saline (pH = 7.6) and incubated with streptavidin-peroxidase (Dako, 1:100) for 40 min 3, 3- diaminobenzidine (DAB) (Dako Glostrup, Denmark) was used as the chromogen; to counteract the core of Gill’s hematoxylin (Polysciences, Warrington, PA, United States) after sections have been dehydrated and assembled. The sections were also incubated with a primary polyclonal rabbit antibody against CD20 (1:1,000; PA5-16701 Thermo Fisher Scientific, Fremont, CA) targeting dog. We do also have evidence of negative controls, not immunoassay and positive control of not tumor cells from the canine that show the sporadic area of immunostaining (Zizzo et al., 2019).

The histologic evaluation was performed independently by 2 of the authors (N.Z and. G.R.). The sections were examined at first with a magnification of ×200 (i.e., ×20 objective and ×10 ocular lens; 0.7386 mm^2^ per field). Subsequently and a 400 × field (i.e., ×40 objective and ×10 ocular lens; 0.1885 mm^2^ for field). Start the count from the top right of all the colored sections, moving down and to the left. The first 10 fields encountered were evaluated for the staining of 50 cancer cells positive and macrophages positive for the most colorful ones. The cell counts positively stained were performed at fields ×400 magnification (Ranieri et al., 2002; Zizzo et al., 2010, 2019). An image analysis system was used were evaluated tumor-associated macrophages (TAMs), lymphocytes, and cancer cells. The association between cells counts and the histological degree was evaluated using the student t-test.

### Inflammatory Cells and Adverse Reactions in EudraVigilance

The method used for data extraction has been used to use the European EudraVigilance database (http://www.ADRreports.eu/it/index.html) which reports spontaneous reports grouped by equipment and systems (S.O.C.) and by subsequent levels and Preferred Terms (P.T.) detected in the EU countries and not EU by healthcare personnel, pharmaceutical companies, and patients. We have focused on the following three mabs: trastuzumab, trastuzumab emtansine, and pertuzumab, proceeding with the search for “reports of suspected adverse reactions by the generic name” within the database. Data on the recently registered drug trastuzumab deruxtecan in which the portion of the IgG1 is linked to an anti-topoisomerase I inhibitor are currently not reported in EudraVigilance to allow a comparative evaluation between mabs.

After selecting the drug of interest, we collected the total number of ADR It is related to it, focusing in particular on the age group between the ages of 18 and 64. The reason for this is the lower presence of confusing factors in this age group, as most young adults and with fewer other therapies in place and co-morbidity. Since, moreover, we have focused our study on drugs used in the treatment of breast cancer, the sex of interest is female, given the higher incidence of this tumor in women. After collecting the number of ADR total per drug from the EudraVigilance database, we looked for the preferred terms (P.T.) of cardiac and vascular reactions related to immuno-inflammatory reactions.

For the reactions S.O.C. “General disorders and administration site conditions” we have selected the most frequently observed such as edema, inflammation, erythema, pain, itching, vasculitis. Continuing with our research, always in this S O.C., we have also searched the database for existing cases of treatment failure, ineffectiveness, non-response or partial response to it, cases of patient non-compliance that may have led to intolerances or dose reduction or even withdrawal.

For the reactions of the “Immune-system disorders” we have focused on the “cytokine release syndrome.”

The P.T. of the S. O. C. “product defects” were reported.

We additionally investigated in the “line listing” section of the EudraVigilance database the trastuzumab ADR filtered by subcutaneous and intravenous administration and analyzed the data in a disaggregated manner in the period 2015–2020, for aged 18–64 years populations. For trastuzumab, there is the intravenous (i.v.) and subcutaneous (s.c.) route of administration, while for trastuzumab emtansine, and pertuzumab only the intravenous one.

The data were exported on the Excel spreadsheet and analyzed. This allowed us to calculate PRR (proportional reporting ratio), a parameter that is, used to calculate the relative risk of ADR within a therapeutic class or related to pharmaceutical classes. PRR greater than 1 indicates a risk of Adverse Drug Reactions (ADR) reported for individuals taking the drug of interest. PRR greater than 1 may also reflect sample changes in data, signaling errors, distorted reports, multiple reports of the same case.

## Statistical Analysis

The data were expressed as mean ± E.S. unless otherwise specified. The significance between data pairs was calculated by the paired *t-student test*. Significant differences of *p* ≤ 0.05 were taken into account. The ADR data extracted from EudraVigilance were collected and analyzed on Excel Software Microsoft.

The PRR was calculated using the following equation:

a/(a+c)/b/(b + d)

where a is the reaction of interest to a given drug of interest, b is the reaction of interest for all other drugs in the class, c are all other reactions to a given drug of interest, d all other reactions to all other drugs in the class (Evans et al., 2001). Signal definition: PRR ≥2, a minimum of three ratios/cases for the reaction of interest, X^2^ ≥ 4. No signal is identified, if PRR is = 1. The duplication of a specific report has been evaluated manually and excluded by the analysis (Mele et al., 2014; Maqoud et al., 2021).

## Results

### Immunohistochemical Analysis of Canine Breast Cancer

The samples under evaluation included 101 tumors of mammary glands. The most frequently were simple carcinoma N = 85 (84%) followed by comedocarcinoma (6.65%), carcinoma–micropapillary invasive (1.9%), carcinoma–anaplastic (2.97%), carcinoma solid (0.95%), and intraductal papillary carcinoma (3.8%). Simple carcinomas were sub-classified to ca. tubule-papillary; cystic-papillary, tubular, and ca. cribriform (Table 1).

**TABLE 1:** Frequency of type of 101 canine mammary tumors in Puglia.

Carcinoma types	Total number of cases
Carcinoma Simple	
Tubulopapillary	37
Cystic-papillary	41
Tubular	5
Cribriform	2
Comedocarcinoma	7
Carcinoma–micropapillary invasive	2
Carcinoma-anaplastic	2
Intraductal papillary carcinoma	4
Carcinoma solid	1

The grading of the carcinomas was as follow out of N = 45 grade I, N = 34 grade II and N = 22 grade III (Table 2).

**TABLE 2:** Frequency of malignant grade of type of 101 canine mammary tumors in Puglia.

Carcinoma types	Grade I	Grade II	Grade III
Carcinoma Simple			
Tubulopapillary	21	8	5
Cystic-papillary	19	12	7
Tubular	4	1	—
Cribriform	—	2	
Comedocarcinoma		2	5
Carcinoma–micropapillary invasive	—	—	2
Carcinoma-anaplastic	—	—	2
Intraductal papillary carcinoma	3	1	
Carcinoma solid		1	
Total	45 (44.5)	34 (33.6)	22 (21.7)

**FIGURE 1:**
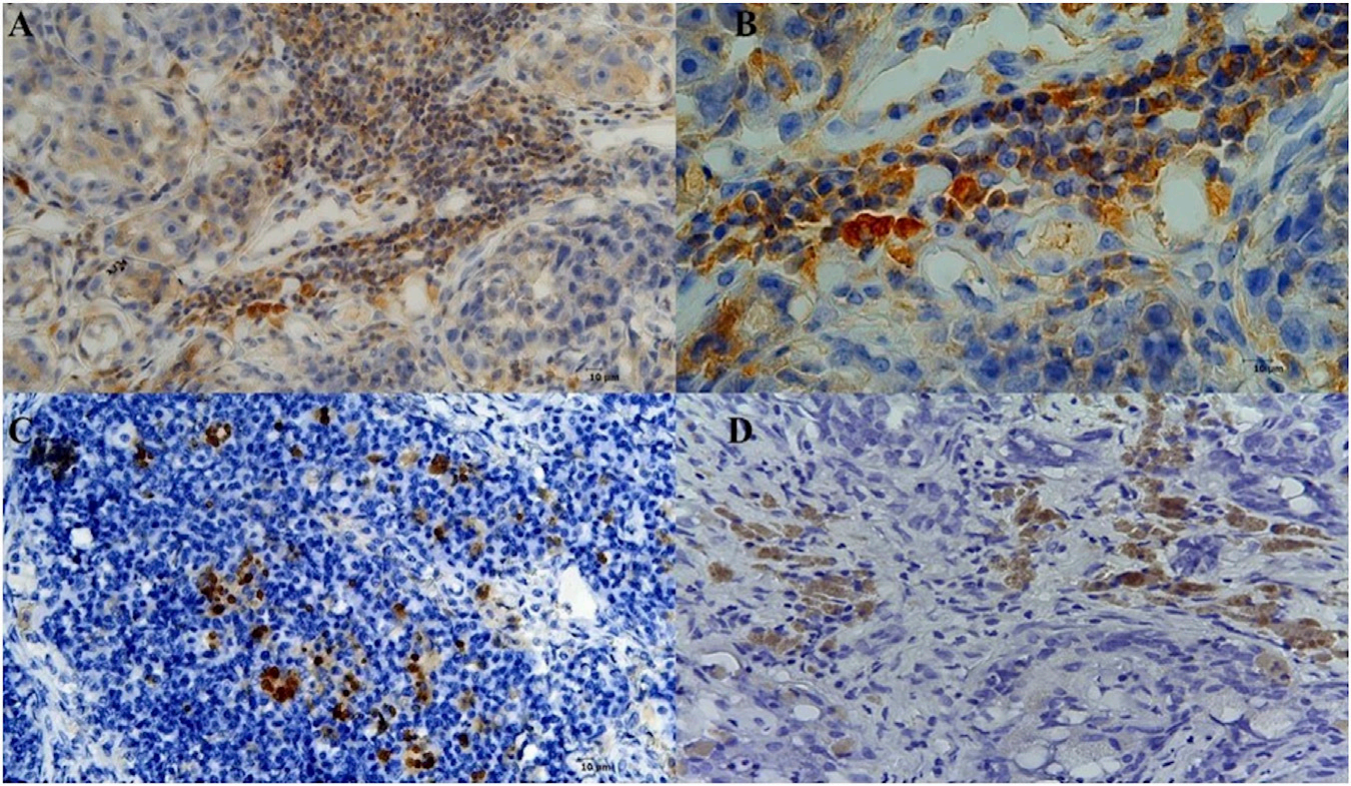
Sections of immunostaining with a monoclonal antiCD20 + mab of canine sections from animals affected by breast cancer. **(A)** CD20 + Lymphocyte infiltrating tumor sample (20X) in a G1 sample; **(B)** CD20 + Lymphocyte infiltrating tumor sample (40X) in a G1 sample; **(C)** CD68 + macrophages infiltrating into the lymph node (20X) in a G3 sample; **(D)** CD68 + macrophages infiltrating into the lymph node in a G3 sample.

**FIGURE 2:**
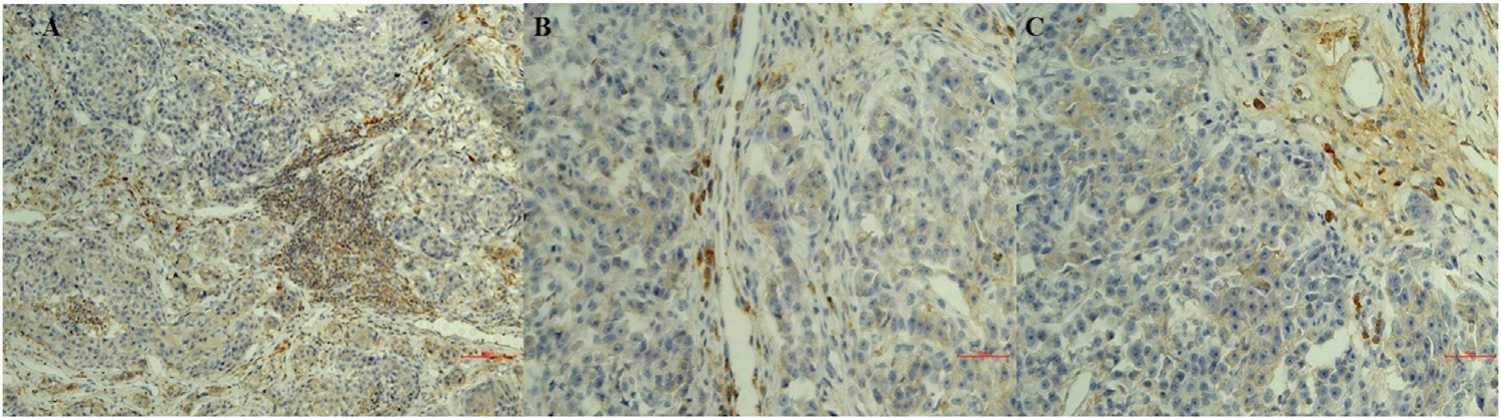
Sections of immunostaining with a polyclonal anti-CD20^+^ mab of canine mammary sections from animals affected by breast cancer. **(A)** CD20 + Lymphocyte infiltrating tumor sample (20X) in a G1 sample; **(B)** CD20 + Lymphocyte infiltrating tumor sample (20X) in a G2 sample; **(C)** CD20 + Lymphocyte infiltrating tumor sample (20X) in a G3 sample.

Infiltrates of CD20 + lymphocytes were also found in tumor samples (Figures 1A,B). A high density was counted in the G1 samples. A decrease in the number of CD20 + lymphocytes was observed with the histological degree of tumors (Figures 2A–C; Table 3). The presence of macrophages was highlighted with the CD68 positive reaction. The density of macrophages in the various types of breast cancer varied from 2 to 71 (average) with ×400 magnification. They were scattered intratumorally, peritumorally, around tumor necrosis and in lymph nodes (Figures 1B,C).

**TABLE 3:** Macrophages and lymphocytes as a function of malignancy grade of canine breast cancer.

Tumor mammary grade (number of cases)	Number of macrophages 400× (0.19 mm^2^)	Number of CD20+lymphocytes 400× (0.19 mm^2^) monoclonal mab	Number of CD20+lymphocytes 400× (0.19 mm^2^) polyclonal mab	Serum platelet lymphocyte Neutrophil/μL (number of cases)
G1 (45)	6 ± 4.24	39.5 ± 10.2	48.1 ± 11.2	221.000 ± 20.000 (4)
	G1 vs. G2 *p* > 0.05 G1 vs. G3 *p* < 0.05	G1 vs. G3 *p* < 0.05	G1 vs. G3 *p* < 0.05	4001.1 ± 800
				5432 ± 1.000
G2 (34)	26 ± 18.38	34.13 ± 9.1	42.13 ± 9.1	295000 ± 30.000 (4)
				2060 ± 600
				8692 ± 432
G3 (22)	46.5 ± 24.64	30.13 ± 4.1	36.3 ± 8.5	381.000 ± 40.000 (4)*
				2721.1 ± 1.100
				11.200 ± 6.300*
				G3 vs G2 and G1*
				*p* < 0.05

Data are expressed as means + standard deviations. Data were significantly different for *p* < 0.05 as determined by the Student t-test. Cell counts were performed at 400× magnification.

In summary, macrophages count was correlated with the grade of severity of the tumor while lymphocytes infiltration was observed in all tumor sections with either monoclonal and polyclonal anti-CD20 mab immunoreactions with an increase in density in the G1 supporting respectively the negative and positive prognostic role of these cells in breast cancer.

The animal samples in G1 showed a reduction of the serum platelet and neutrophil counts and elevated lymphocyte count, while the animal samples in G3 showed the opposite condition (Table 3).

### PRR of Different ADR of Mabs in the General Population

**FIGURE 3:**
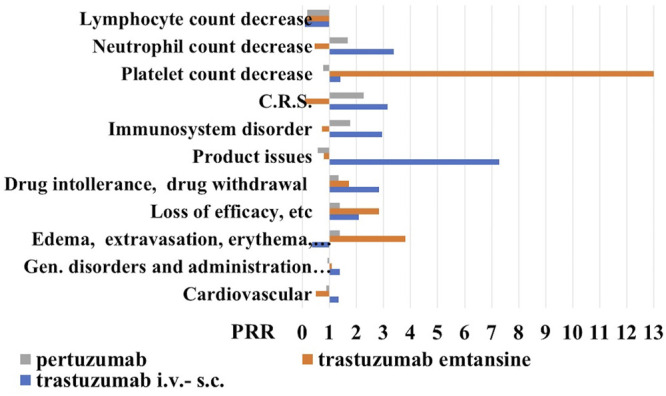
Proportional reporting ratio (PRR) of reactions (all grade) in the 18–64-year-old population under anti-HER2 mabs treatment. Trastuzumab i.v.-s.c. formulations with X^2^ >4.612 for product issues, immune-system disorder, cytokine release syndrome (C.R.S.) and drug intolerance, and trastuzumab emtansine with X^2^ >4.522 for edema, extravasation, inflammation, and loss of efficacy, and pertuzumab. PPR of cell biomarkers of inflammation (all grade) in the 18–64-year-old population under anti-HER2 mabs treatment. Trastuzumab emtansine showed X^2^ >5.121 for lymphocyte and platelet count decrease. PRR >2 indicates a high risk of ADR for a specific S.O.C.

We found that trastuzumab has the highest number of adverse drug reactions of 12711 also related to the historical use of this drug, followed by pertuzumab with 3230 and trastuzumab emtansine with 1345 reports on 04 March 2021. Among the various ADR, trastuzumab showed the highest number of cardiovascular reports with 2858, followed by pertuzumab with 616 and trastuzumab emtansine with the lowest number of reports 151. The calculation of the PRR gave an estimate of about 1 for all mabs indicating a similar cardiovascular profile (Figure 3).

Proceeding with the analysis, we reported the number of reports related to S.O.C. “general symptoms and related to the site of administration” and trastuzumab has the largest number of cases of 3605, compared to pertuzumab with 880 reports and trastuzumab emtansine with 414 reports. The P. R. R., in this case, remains around the unit for all mabs. However, going into the detail of this S.O.C., we have noticed that trastuzumab presents 23 cases (to date) of edema, erythema, itching, pain, inflammation, and local reactions related to the use of venous catheters, compared to the other two drugs, for which we have recorded 11 cases each. Here the PRR, however, remains under the unit for trastuzumab, just over the unit for pertuzumab, and being higher for trastuzumab emtansine (Figure 3). Trastuzumab has also 195 cases of loss of efficacy and ineffectiveness compared to 58 reports of trastuzumab emtansine and 66 of pertuzumab, data that increased the PRR values much higher than the unit for the trastuzumab emtansine. Considering drug intolerance and possible withdrawal, we found 40 cases for trastuzumab, 7 for emtansine, and 12 for pertuzumab, again the P. R. R. values were higher than the unit for all three mabs, with trastuzumab showing a PRR value >2, unlike for trastuzumab emtansine.

Moving on to the S.O.C. immune system reactions, we found 38 for trastuzumab emtansine, 178 reports for pertuzumab, and 487 cases for trastuzumab. The analysis of PRR reveals that trastuzumab emtansine has a higher relative risk of adverse reactions of the immune system, at least compared to the other two mabs, since with different mechanisms of action. We have searched the cytokine release syndromes and some interesting data have emerged: first of all, trastuzumab emtansine has no such case to date; pertuzumab showed 3 cases and of the 7 cases reported for trastuzumab, almost half were male with P. R. R. values of the trastuzumab >3.

For the S. O. C. “product defects” we found 43 reports for trastuzumab with a PRR value >7, 3 reports for trastuzumab emtansine, and 5 reports for pertuzumab (Figure 3).

### Investigation on Inflammatory Biomarkers in General Population

We found that trastuzumab i.v.-s.c. (3435) showed 39 (1.11%) reports of C reactive protein increase, 14 (0.4%) of eosinophil count increase, 32 (0.93%) reports of lymphocyte count decrease of which 17 reports were associated with the s.c. formulation, but an elevated number of reports of neutrophil count decrease of 363 and platelet count decrease of 258 representing however respectively the 10.5 and 7.5% of the blood and lymphatic system disorder reactions.

Trastuzumab emtansine (623) was associated with lymphocyte count decrease reports of 9 (1.4%), neutrophil count decrease of 21 (3.37%) and C reactive protein increase of 6 (0.96%), and an elevated number of platelet count decrease of 286 reports (45.9%).

Pertuzumab (1122) showed 128 reports of the neutrophil count decrease representing 11.4% of blood and lymphatic system disorder reactions, platelet count decrease of 69 (6.14%), lymphocyte count decrease of 19 (1.6%), and C reactive protein increase of 10 (0.89%).

Trastuzumab i.v-s.c. and trastuzumab emtansine showed the highest PRR values >2 for neutrophil and platelet count decrease, respectively, in the absence of reports of lymphocyte decrease (Figure 3).

Based on our data, a low neutrophil-to-lymphocyte and platelet-to-lymphocyte ratios can be therefore expected in the general population respectively treated with trastuzumab i.v.-s.c. and trastuzumab emtansine i.v., with a better outcome.

To evaluate this hypothesis, we investigated the possible relationship between the trastuzumab i.v-s.c. (363 reports) and trastuzumab emtansine (286 reports) induced neutrophil and platelet count decrease and disease progression. Case report analysis however failed to correlate disease progression with serious platelet count decrease and serious neutrophil count decrease, which indeed were found respectively in only 3.3 and 0.93% of the case reports of trastuzumab emtansine and trastuzumab i.v.-s.c.

Analyzing, moreover, the ADR for all 4 drugs including the different trastuzumab i.v and s.c. formulations in the 2 years 2019–2020, among adults (18–64), we have noticed the absence of cases of myocarditis for trastuzumab i.v. and s.c., despite the presence in 2020 for the intravenous trastuzumab of 34 cases of left ventricular dysfunction, hypertension, hypotension, arrhythmia and heart failure as expected reactions, with 15 withdrawn in polytherapy and with pre-medications; while 0 cases of the same reactions for the s.c. the formulation in the same year. In 2019, the s.c. formulation reported 1 case among the reactions mentioned above and no withdrawal, while 14 cases for the i.v. and 5 withdrawals. 1 case of pericardial reaction for trastuzumab i.v. was reported in 2020 in polytherapy with docetaxel and 1 withdrawal, while 0 cases for s.c. the formulation in the same year.

Concerning trastuzumab emtansine there were, on the other hand, 2 cases of myocarditis in 2020, while 0 cases in 2019. Also in 2020, 2 cases were reported of expected left ventricular dysfunction, hypertension, hypotension, arrhythmia, and heart failure, plus 1 withdrawal, a situation very similar to 2019. No cases of vasculitis even from trastuzumab emtansine, at least in the 2 years 2019–20.

For pertuzumab 0 cases of myocarditis in 2020, and 21 cases of expected left ventricular dysfunction, hypertension, hypotension, arrhythmia, and heart failure, in polytherapy with 8 withdrawal, and 10 cases of left ventricular dysfunction, hypertension, hypotension, arrhythmia, and heart failure and 4 withdrawn in 2019. In the same year and for the same drug, there were also 2 cases of myocarditis, of which 1 withdrawal. Pertuzumab did not show cases of other pericardial problems in the 2 years 2020–2019. It is reported, instead, among the ADR 1 case of vasculitis in 2020, while 0 cases in 2019.

We found that in 2019 the intravenous formulation of trastuzumab in combination with drugs used as “premedication,” showed in the young adult population (18–64 years) 26 reactions including dyspnea, edema, anaphylactic shock, and hypersensitivity with 8 dose reductions and 14 withdrawn, while these cases were not observed (to date) with the subcutaneous formulation.

In 2020, however, we searched for ADR hypersensitivity and anaphylactic reaction, and 32 reports were reported for the intravenous trastuzumab with 1 dose reduction and 6 withdrawn. With the subcutaneous formulation, we noticed the report of 1 case of hypersensitivity, but no cases of anaphylactic reactions.

Therefore, rare cases of immunological reactions affecting cardiovascular apparatus were reported with the anti-HER2 mabs in the 2 years of observation.

**FIGURE 4:**
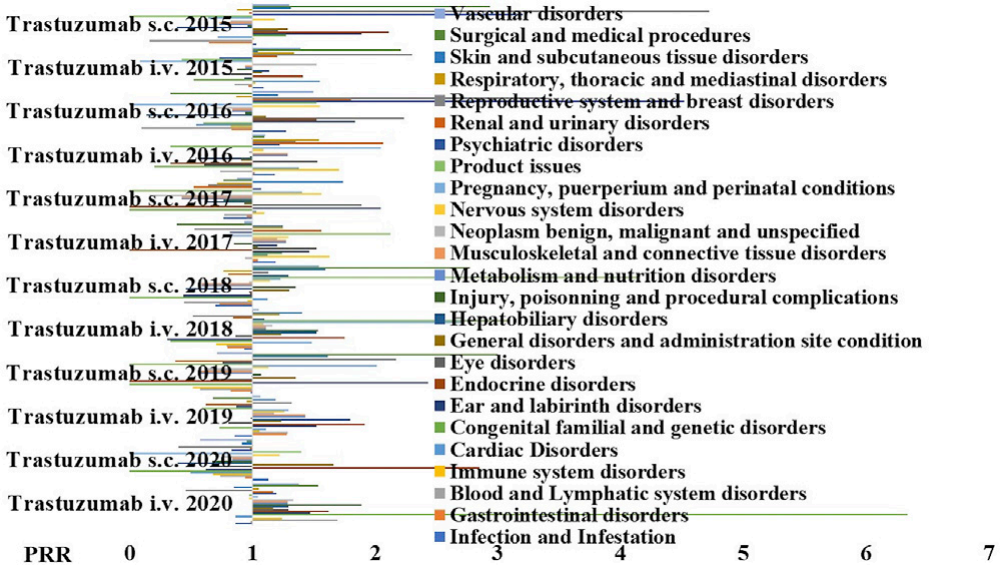
PPR distribution of trastuzumab s.c. and i.v. formulations during the period of observation of 2015–2020. PRR ≥2 indicates a high risk of ADR for a specific S.O.C. Trastuzumab i.v. showed PRR ≥2 for product issues (X^2^ > 4), general disorder and administration site conditions (X^2^ > 4); trastuzumab s.c. showed PRR ≥2 general disorders and administration site condition (X^2^ > 4), product issues (X^2^ > 4), infection and infestation (X^2^ < 4, n.s.); musculoskeletal and connective tissue disorders (X^2^ > 4), and psychiatric disorders (X^2^ > 4).

We further investigated the reactions that may also have an inflammatory pathophysiological basis reported for the s.c and i.v formulations of trastuzumab to further evaluate the contribution of the administration route and the PRR were calculated (Figure 4), and we found an unbalance for the S.O.C. Musculoskeletal and connective tissue disorders in particular for the arthralgia that was reported mostly in patients treated with the s.c. formulation with severe cases that led to drug withdrawal (Table 4).

**TABLE 4:** Cases of “Musculoskeletal and connective tissue disorders” of the intravenous and subcutaneous trastuzumab (2015–20).

Drug	N (%)	Reaction	Concomitant drug	Drug withdrawn
Trastuzumab s.c. 2017	52 (9.84%)	Back pain Arthralgia Pain in extremity Neck pain	Docetaxel Paclitaxel Valsartan, Hydrochlorothiazide Atenolol Exemestan Letrozole Dexamethason Lansoprazol Furosemide	13
Trastuzumab s.c. 2016	113 (9.33%)	Arthralgia Asthenia Back pain Pain in extremity	Docetaxel Anastrozole Tamoxifen Exemestan Letrozole Dexamethasone	4
Trastuzumab i.v. 2015	150 (3.42%)	Arthralgia Myalgia Back pain	Dexamethasone Letrozole Docetaxel Paclitaxel Cyclophosphamide Carboplatin	25
Trastuzumab s.c. 2015	58 (10.13%)	Abdominal pain Arthralgia Myalgia Back pain	Carboplatin Tamoxifen	3

An unbalance for the S.O.C. Product issues were also reported mostly in patients treated with the i.v. formulation with severe cases that led to drug withdrawal (Table 5).

**TABLE 5:** Cases of “Product Issues” of the intravenous and subcutaneous trastuzumab (2015–20).

Drug	N (%)	Reaction	Concomitant drug	Drug withdrawn
Trastuzumab i.v. 2018	17 (0.37%)	Product substitution issue alanine aminotransferase increased aspartate aminotransferase increased blood bicarbonate decreased blood alkaline phosphatase increased Product quality issue	Pertuzumab Denosumab Dexamethasone Celecoxib	4
Trastuzumab s.c. 2018	4 (0.66%)	Infusion-related reaction product complaint dehydration	Not reported	1
Trastuzumab i.v. 2017	15 (0.27%)		Paclitaxel Pertuzumab Capecitabine Enalapril Fosphomicyn Levothyroxine Lyrica Metamizole sodium	6

An unbalance for the S.O.C. Psychiatric disorders were also reported in the patient population treated with the s.c. formulation (Table 6).

**TABLE 6:** Cases of “Psychiatric disorders” of the intravenous and subcutaneous trastuzumab (2015–20).

Drug	N (%)	Reaction	Concomitant drug	Drug withdrawn
Trastuzumab s.c. 2016	68 (5.61%)	Psychotic disorder confusional state anxiety asthenia depression and arthralgia blood pressure increased cough	Amitriptyline letrozole codeine atorvastatin desorgestrel docetaxel ramipril tamoxifen rivaroxaban	2
Trastuzumab s.c. 2015	25 (4.37%)	Anxiety asthenia crying insomnia arthralgia	Bupropion docetaxel colestyramine	1

## Discussion

In this work, we investigated the role of inflammatory cells experimentally in a spontaneous canine model of breast cancer. The role of the inflammatory cells was also evaluated using data from the spontaneous report in the human database. Inflammatory and immune-related adverse reactions (ADR) in breast cancer were extracted from EudraVigilance in the unselected adult population (18–64 years.o.) under anti-HER2 treatments.

Trastuzumab i.v.-s.c. showed a low risk of cardiovascular and inflammatory reactions (PRR < 2) and a PRR ≥2 for loss of effectiveness, but unfavorable PRR values > 2 for immune system reactions and intolerances with drug withdrawal, and a PRR >7 for technological problems mostly related to the use of the i.v. device.

Trastuzumab emtansine showed a high risk within the mabs of loss of effectiveness with a PRR >2 and an unfavorable PRR >3 for inflammatory reactions with edema, skin reactions, and extravasation mostly due to the serious grade of reactions (PRR > 3), but a more favorable PRR values <1 for the cardiovascular and immune system within the mabs. The different safety profile of this mab vs. the trastuzumab i.v.-s.c. can be ascribed to the presence of the emtansine inducing inflammatory reactions.

Pertuzumab showed a low risk of loss of efficacy with a PRR <2, favorable PRR values for C.R.S., immunological and inflammatory reactions due to not serious grade of reactions (PRR < 1), and values <1 for cardiovascular and other reactions.

Inflammatory cells have a recognized role in canine breast cancer like in humans (Carvalho et al., 2014). We found that macrophages count was correlated with the grade of severity of the tumor in canine breast cancer and lymphocytes infiltration were observed in all tumor sections however showing an inverse correlation with the grade of severity of the tumor thereby increasing in density in the G1 supporting respectively the negative and positive prognostic role of these cells in breast cancer also in the dog. B lymphocytes do produce cytokines that support the T cell response promoting the CD8^+^ lymphocytes cytotoxic activity. The B cells may also act as antigen-presenting cells to T cells, and produce antibodies directed against tumor antigens. B lymphocyte-derived antibodies have been shown to recognize tumor antigens per se.

Inflammatory and immune-related adverse reactions were associated with the use of mabs with pertuzumab showing a low risk of loss of efficacy and C.R.S., immunological, inflammatory reactions and cardiovascular reactions, and low risk of reduced inflammatory cell counts. Inflammatory reactions with edema, skin reactions, and extravasation mostly due to serious grade of reactions and a high number of platelet count and lymphocyte count decrease were associated with trastuzumab emtansine that showed a high risk within the mabs of loss of effectiveness, but a favorable profile for the cardiovascular and immune system within the mabs. A case report showed the first case of trastuzumab emtansine-associated pleural and pericardial effusions in a patient with breast cancer in the absence of other etiologies (Lombardi et al., 2021), and higher rates of peripheral neuropathy and liver disfunction was also reported following trastuzumab emtansine treatments in the patient’s population (Battisti et al., 2020). Trastuzumab i.v.-s.c. showed however a low risk of cardiovascular and inflammatory reactions as well as of loss of effectiveness, but unfavorable risk profile for immune system reactions and intolerances and technological problems. This is associated with platelet count and neutrophil count decrease reports.

Trastuzumab i.v.-s.c. and trastuzumab emtansine respectively showed a high neutrophil count decrease reports and a high platelet count reports of serious grade in the absence of lymphocytes changes not associated with disease progression as by case report analysis thereby indicating a better outcome.

Analyzing, moreover, the ADR for all 4 drugs including the different trastuzumab i.v and s.c. formulations in the 2 years 2019–2020, among adults (18–64), we have noticed the absence of cases of myocarditis for trastuzumab i.v. and s.c., 2 cases of myocarditis for trastuzumab emtansine and pertuzumab, and no case of vasculitis for trastuzumab emtansine and 1 case for pertuzumab. Therefore, rare cases of immunological reactions affecting cardiovascular apparatus were reported with the anti-HER2 mabs in the 2 years of observation.

We further investigated the reactions that may also have an inflammatory pathophysiological basis reported for the s.c and i.v formulations of trastuzumab to further evaluate the contribution of the administration route, and we found that trastuzumab s.c. showed additional risk related to infection and infestation, musculoskeletal and connective tissue disorders with arthralgia, and psychiatric disorders. Clinical Trials (C.T.) showed no differences in terms of efficacy between the two formulations of trastuzumab but safety was in favor of i.v. formulation vs s.c. formulation to date with an unbalance of serious A.E. of the s.c. formulation (Ismael et al., 2012; Gligorov et al., 2017; Jackisch et al., 2019).

We found several similarities in our investigation using pharmacovigilance data with C.T. data; severe cases of arthralgia, asthenia, and fatigue were more frequently reported in the patients treated with the s.c. formulation of trastuzumab. Arthralgia, diarrhea, and fatigue were indeed the most commonly reported AEs in HER2-positive breast cancer patients under treatment with s.c trastuzumab in combination with chemotherapy (Ismael et al., 2012; Gligorov et al., 2017). In addition, we found an unbalance related to the infections and infestations again in favor of i.v formulation like in C.T. trials. No differences were found in the cardiovascular safety between the two formulations in our investigations in agreement with C.T. data. Differently from C.T. data, we did not find reports on anti-drug antibody production or PH-20 antibodies against the human hyaluronidase as instead demonstrated in the open-label Phase 3 HannaH trial (Jackisch et al., 2019).

We additionally found an imbalance of psychiatric disorders in the patients treated with the trastuzumab s.c. vs. the i.v. with concomitant use of antipsychotic and anxiolytic drugs that were not reported in C.T. that can be related with the use of a novel unknown formulation. Depressive disorders were on the other hand observed in these patient populations (Chang et al., 2015).

Despite these reports and the not favorable cost (Inotai et al., 2019), it is of note the fact that several patients have a great benefit from the trastuzumab s.c. the formulation in terms of quality of life and tolerance particularly those under long-term maintenance therapy that are not anymore exposed to chemotherapy. It is of note the fact that the FDA’s approved in 2020 following the FeDeriCa study (https://www.notiziariochimicofarmaceutico.it/2019/12/20/formulazione-sottocutanea-di-pertuzumab-e-trastuzumab-per-il-tumore-al-seno-her2-positivo) the combo pertuzumab + trastuzumab s.c. that is, now available.

## Conclusion

Pharmacovigilance data combined with experimental data using a spontaneous animal model of disease sharing the same environment of human patients offered the advantage of evaluating the role of the inflammatory reactions and related cells in breast cancer patients under mabs treatment in uncontrolled conditions avoiding the possible bias of data selection. The finding that trastuzumab i.v-s.c. and trastuzumab emtansine showed, respectively, high neutrophil and platelet count decrease with no cancer progression in the absence of reports of lymphocyte decrease, and that a high density of CD20^+^ lymphocytes was observed in the less severe canine breast cancer samples in parallel with high neutrophil and platelet count decrease support the idea that these cells play a favorable role either in breast cancer in human and in this spontaneous model of disease.

## Data Availability

The original contributions presented in the study are included in the article/supplementary material, further inquiries can be directed to the corresponding author.
